# A Psychoeducational Workshop for the Parents of Young Voice Hearers: A Preliminary Investigation into Acceptability and Outcomes in an NHS Child and Adolescent Mental Health Service

**DOI:** 10.1177/13591045231167969

**Published:** 2023-06-05

**Authors:** Annabelle Deane, Lyn Ellett, Mark Hayward

**Affiliations:** 1 School of Psychology, 3162 Royal Holloway University of London, London, UK; 2 School of Psychology, 7423 University of Southampton, Southampton, UK; 3 R&D Department, Sussex Education Centre, Sussex Partnership NHS Foundation Trust, Hove, UK; 4 School of Psychology, 1948 University of Sussex, Brighton, UK

**Keywords:** hearing voices, auditory hallucinations, young people, parents, psychoeducation

## Abstract

Background: Hearing voices is a common experience in young people and can be associated with distress, self-harm, and an increased risk of attempting suicide. Many parents lack confidence in supporting young people who are distressed by voices. However, there are currently no evidence-based interventions to support the parents of young voice hearers. Method: This was an uncontrolled study exploring the preliminary acceptability and outcomes of a psychoeducation workshop for the parents of young people experiencing distressing voices within a Child and Adolescent Mental Health Service (CAMHS) in the UK’s National Health Service. Results: A total of 21 parents consented to participate, 15 attended a workshop and 10 provided complete data sets. Five workshops were delivered across a seven-month period. Qualitative feedback was suggestive of acceptability and highlighted possible adaptations in relation to inclusivity, content focus and delivery format. Participants reported increased confidence and improved attitudes and beliefs towards voice hearing. Conclusions: The findings from this study suggest that a psychoeducational workshop within a CAMHS context can be acceptable and helpful for parents of young people with distressing voice hearing experiences. Adaptations to the workshop are required to maximise inclusion, engagement, and outcomes.

## Introduction

Hallucinations can be experienced within any of the five senses and are reported by clinical and non-clinical populations (Jardri et al., 2014). When experienced in the auditory modality, hallucinations (also referred to as hearing voices) happen when an auditory experience (e.g., noises, voices, other audible perceptions with verbal content, etc.) occurs in the absence of any corresponding sensory input (Jardri et al., 2014). This is a common experience for young people, with prevalence rates estimated at 12% (Maijer et al., 2017). Voices in this population have been shown to be experienced as very critical (e.g., ‘They can make me feel bad and tear me down’; Parry & Varese, 2021a), domineering (e.g., ‘They constantly tell me to harm myself’; Parry & Varese, 2021b) and disruptive (e.g., ‘… I find it hard to concentrate when they talk a lot as I get pulled into what they are saying and find it hard to hear people around me.‘; Parry et al., 2020). These experiences can have many negative impacts on young people (e.g., ‘It has completely changed me. It’s made me feel really crap. The voices don’t leave me alone. I’ve lost a lot of things … I ain’t got a life any more really. I can’t go out’; Denno et al., 2021).

Hearing voices in young people has been associated with an increased risk for suicidal ideation and plans (odds ratios [OR] of 1.9 and 2.2, respectively), and suicide attempts (OR 3.99; DeVylder & Hilimire, 2015). Amongst young people who were experiencing suicidal ideation, hearing voices was associated with increased risk of suicide attempts (OR 3.4; Fujita et al., 2015). Voice hearing has also been associated with self-harm (OR 4.87; Hielscher et al., 2018), with increased risks not attributable to co-occurring mental health problems (Maijer et al., 2019). When voice hearing persists for young people, this can be indicative of severe mental health problems in the future (Bartels-Velthuis et al., 2016), including psychosis (OR 16.4; Poulton et al., 2000).

Young people, clinicians and parents can be reluctant to talk about voice hearing (Bogen-Johnston et al., 2019; Parry & Varese, 2021a; Rammou, 2021). Qualitative studies have identified barriers to the disclosure of voices by younger people, including stigma and shame, threats from voices and the possibility of a negative response from other people (Bogen-Johnston et al., 2019). A clear need has been identified for structured interventions to support clinicians to start and continue conversations about voices with young people (Bogen-Johnston et al., 2020). Additionally, parents have reported feeling unprepared to help and support their child, feeling isolated and unsupported by a spouse and mental health services, and expressed concern that seeking help could exacerbate the child’s anxieties (Parry & Varese, 2021a). Solutions recommended by these parents included the provision of inclusive community and school-based family support and earlier intervention pathways, and interventions that attend to the building of coping skills and enhance parental wellbeing.

There are currently no evidence-based interventions for the treatment of distressing voices for young people. A focus upon coping strategies has been suggested by young people as a useful approach for intervention (Denno et al., 2021). Promising interventions in this respect include the Stronger Than Your Voice intervention (coping and psychoeducation; Maijer et al., 2020) and Coping Strategy Enhancement (CSE; Hayward et al., 2022). Interventions are also required to enhance parental knowledge and confidence, to enable parents to support the use of coping strategies by their children. However, there are no known interventions for parents in the published literature. The aim of this study was to conduct a preliminary investigation of the acceptability and outcomes associated with a psychoeducational workshop for the parents of young people who were distressed by hearing voices.

## Method

### Study Design

This study was delivered in the Sussex Voices Clinic (SVC), a trans-diagnostic outpatient service for voice hearing offered to young people and adults within routine clinical services in an NHS Mental Health Trust in Sussex, UK. The study used an uncontrolled design. Quantitative and qualitative data addressing acceptability of the workshop were collected at post workshop (T1) and quantitative data addressing outcomes were collected at pre-workshop (T0), post-workshop (T1) and 4-week follow up (T2).

### Participants

Parents of young voice hearers accessing the SVC were invited to take part. Inclusion criteria specified that participants were a primary caregiver for a young person (<18-years-old) who heard voices and was accessing the SVC, were aged 18 or over, had the ability to speak and read English fluently, and had access to the Internet.

### Lived Experience Involvement

Two mothers and one grandmother of young people who had attended the SVC were contacted to offer their consultation on the study. One was able to offer their consultation, one had prior commitments, and one did not respond. Prior to seeking ethical approval, a meeting was held between the consultant and lead researcher. Outcome measures and study materials were examined for their accessibility to a lay audience and alternative language was used where required. The consultant also advised on which items should be included within the Attitudes and Beliefs towards Voice Hearing outcome measure.

### Measures

#### Participant Experience Questionnaire

Acceptability was measured using a 14-item experience questionnaire, adapted from Jolley et al.’s (2015) Patient Experience Questionnaire (PEQ). The first six items were measured on a four-point Likert scale, from 0 (rarely/never) to 3 (at all times). Example items included “Do you feel that the workshop has helped you to better understand and address your child’s difficulties?” and “Did the workshop materials (e.g., slides and handouts) make sense?“. These six items were analysed individually. The next six questions were open-ended and invited participants to elaborate on the overall experience, and positive and negative aspects of the workshop, alongside any further comments and suggestions. The final two questions included “How likely would you be to recommend the Sussex Voices Clinic workshop to friends and family if they needed similar support for their young person?” measured with a 6-point Likert scale from “Extremely Likely” to “Extremely Unlikely” and “Overall, how would you rate your experience of the Sussex Voices Clinic workshop?” measured on a 10-point Likert scale from 1 (very poor) to 10 (excellent).

### Outcomes

#### Attitudes and Beliefs towards Voice Hearing (ABVH)

Eleven items were selected from an existing 35-item scale measuring attitudes and beliefs towards voice hearing amongst clinicians (Rammou, 2021). Wording was changed from clinician to parent. All items were scored on a 7-point Likert scale from 1 (Strongly disagree) to 7 (Strongly agree) and participants could score between 11 – 77, with higher scores reflecting more positive attitudes and beliefs towards supporting a young person hearing voices. Internal consistency for the scale at baseline was in the ‘questionable’ range (*N* = 20, α = .66) after the item “I feel I have the right to ask a young person questions about their distressing voice-hearing when necessary” was removed (α = .61 for 11 items).

#### Self-Efficacy

A three-item scale by Rammou (2021) measuring the self-efficacy of clinicians working with young people who hear voices was used with wording changed to address parents. Participants were asked to rate their degree of confidence towards three statements (‘Ask a young person if they hear voices’, ‘Discuss voice-hearing experiences with a young person who hears distressing voices’, and ‘Provide useful information (e.g., educational material and tips to manage voices) to a young person who hears distressing voices’) on a sliding scale from 0 (Cannot do at all) to 100 (Highly certain can do). In line with Rammou (2021), the three self-efficacy items were analysed and reported separately, as they were considered to measure distinct constructs.

### Procedure

Ethical approval was granted by the Health Research Authority (reference 21/WM/0098) and the Royal Holloway University of London Research Ethics Committee (REC Project ID: 2608); the study was also approved by Sussex Partnership NHS Foundation Trust's Research and Development Department.

All participants read an information sheet and provided written informed consent. Participants completed all questionnaires independently via Qualtrics links that were emailed a week before the workshop (T0), on the day of the workshop (T1) and 4 weeks after the workshop (T2). They completed the workshop on Zoom. All participants completed T0 questionnaires before the workshop. Thirteen participants completed T1 questionnaires within a week after the workshop and one participant within 9 days. Seven participants completed T2 questionnaires within a week of receiving them and one participant within 1 month. Participants were debriefed at the end of the study.

### Workshop Content

Two-hour online workshops were facilitated by a Clinical Psychologist and a Clinic Assistant from the SVC, and a CAMHS Psychiatrist. Workshops consisted of three main components: (1) psychoeducation about voice hearing; (2) an overview of the CSE intervention (Hayward et al., 2022) and how it can be used to support young people, and (3) tips on talking about voices with a young person.

#### Psychoeducation

After introductions, participants were provided with 12 ‘fact or fiction’ statements about voice hearing. The statements encouraged participants to reflect on their existing knowledge and dispel common misconceptions. The knowledge imparted by the statements was reinforced by some statistics on the prevalence of voice hearing across the lifespan.

#### Coping Strategy Enhancement

Participants were introduced to the principles of the intervention that was/would be delivered to their young person within SVC. Discussions focused upon: what happens before voices (triggers), what happens after voices (helpful and unhelpful responses) and when are voices not around? A case study of a fictional young person was used interactively to apply the principles to everyday experience.

#### Tips for Talking About Voices

The final part of the workshop covered tips for talking about voices with a young person. These tips were taken from the chapter for carers within the CBT self-help book ‘Overcoming Distressing Voices’ (Hayward et al., 2018). Emphasis was placed upon acceptance, being non-judgmental, building warmth and space, and being curious. Tips from young voice hearers about what they themselves found helpful were also included. Finally, participants were offered a free copy of the book ‘Overcoming Distressing Voices’. The book was sent to participants immediately after the workshop.

### Data Analysis

Sociodemographic characteristics of participants and young people were summarised by count (*n*), percentage (%), mean (*M*) and standard deviation (*SD*).

### Acceptability

Quantitative data collected on the PEQ was reported as percentages of responses to Likert-style questions and mean and standard deviation of scaled questions. Qualitative data from open-ended questions on the PEQ were analysed through a directed content analysis (Hsieh & Shannon, 2005), using categories extracted from a literature review on parental engagement in interventions for young people with mental health difficulties (Deane, 2022). Responses were coded line by line. A unit of analysis was defined as any length of text which captured the pre-existing framework, ranging from a few words to a sentence. The procedures recommended by Hayes and Krippendorff (2007) for inter-coder reliability were calculated between two independent coders based on 80% of all raw data with a kappa value of 0.89, indicating strong reliability.

### Outcomes

Self-efficacy and ABVH outcomes were reported as mean (*M*), minimum and maximum values, standard deviation (*SD*), and 95% confidence intervals (CI) of the unstandardised effect at T0, T1 and T2. Due to the small sample size <30, *t* scores were used to calculate 95% CIs (Rumsey, 2016). Effect sizes were reported as Cohen’s *d*_*av*_ as suggested to calculate Cohen’s *d* for within-subjects designs (Lakens, 2013). These were interpreted according to Cohen's (1988) guidelines of small (*d* = 0.2), medium (*d* = 0.5), and large (*d* ≥ 0.8). Missing data was assessed, however there were no attempts to replace it (as in previous research e.g., Hayward et al., 2022).

## Results

### Participant Flow

**Figure 1.**
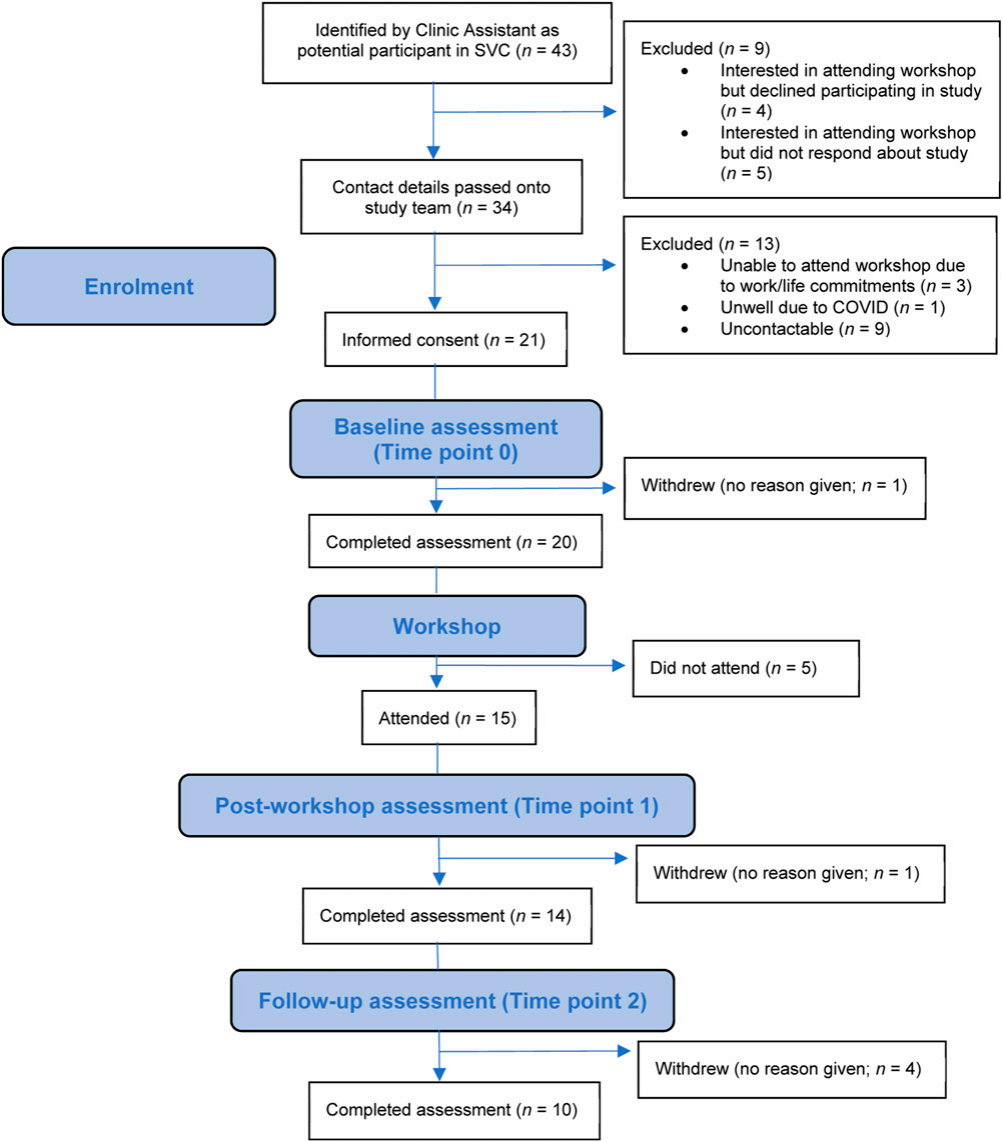
CONSORT flow diagram.

Participants were recruited between June and November 2021. Forty-three potentially eligible parents were identified through the SVC. Of those parents, 21 (48.8%) gave consent to take part in the study. See Figure 1 for a CONSORT diagram illustrating the flow of participants through the study. The status of the young people related to the participants were as follows: awaiting assessment (*N* = 15); currently in therapy (*N* = 2); completed therapy (*N* = 2); waiting for therapy (*N* = 1); assessment indicated therapy was not suitable (*N* = 1).

### Participant Characteristics

Sociodemographic data was provided by 19 out of 21 participants who provided consent, all of whom identified as parents. There were 4 male and 15 female participants (M_AGE_ = 47.4 years, *SD* = 5.44, range: 37–54), most of whom identified as White British (17; 1 Chinese/Chinese British; 1 Mixed Ethnicity). Regarding the young person they cared for, 10 (52.6%) were male and 8 (42.1%) were female, (M_AGE_ = 14.7 years, *SD* = 1.66, range: 12 – 17) and most identified as White British (16; 3 mixed Ethnicity).

Five workshops were delivered in total. The average number of participants attending each workshops ranged from one to five. Some parents attended a workshop but did not consent to taking part in the study. The adjusted average number of attendees ranged from two to eight when including these parents. The number of workshop attendees is shown in Table 1.

**Table 1.** Number of Parents Attending Each Workshop.

Workshop	1	2	3	4	5
Participants only	5	4	2	3	1
All attendees^a^	8	6	2	3	3

*Note*. ^a^Represents both participants and parents who attended a workshop but did not participate in the study.

### Acceptability

Fourteen of the 15 (93.3%) participants who attended a workshop also completed the PEQ which assessed the acceptability of the workshop to parents (See Table 2). When asked how likely participants would be to recommend the workshop to family or friends, 85.7% of participants said extremely likely, 7.1% said likely, whilst 7.1% said extremely unlikely. Participants rated their experience of the workshop an average 8.47 (*SD* = 1.93) out of ten.

**Table 2.** Participant Responses on the PEQ.

Item	*M*	*SD*	(1) Rarely/Never	(2)	(3)	(4) At All Times
Did staff listen to you and treat your concerns seriously?	3.73	0.58	0 (0.0%)	1 (7.14%)	1 (7.14%)	12 (85.71%)
Do you feel that the workshop has helped you to better understand and address your child’s difficulties?	3.24	0.94	1 (7.14%)	1 (7.14%)	3 (21.43%)	9 (64.29%)
Did the workshop materials (e.g., slides and handouts) make sense?	3.48	0.84	1 (7.14%)	0 (0.0%)	2 (14.29%)	11 (78.58%)
Was the content of the workshop useful for you?	3.18	0.93	1 (7.14%)	1 (7.14%)	4 (28.57%)	8 (57.14%)
Did you have confidence in your facilitators and their skills and techniques?	3.92	0.27	0 (0.0%)	0 (0.0%)	1 (7.14%)	13 (92.86%)
On reflection, did you get the help that mattered to you?	3.18	0.93	1 (7.14%)	1 (7.14%)	4 (28.57%)	8 (57.14%)

*Note.* All ratings are based on a 4-point scale in order to calculate the mean of frequencies and standard deviation. Each item ranged from 1 (Rarely/Never), 2 (Sometimes), 3 (Most of the Time), to 4 (At All Times).

### Qualitative Data

Data from participants’ responses to five open-ended questions about their satisfaction of the workshop was analysed using directed content analysis with two categories (Hsieh & Shannon, 2005).

### Who Should be in the Workshop Space?

The first category generated responses relating to those who attended the workshop, those who facilitated and those who were absent. In relation to the attendees, the value of meeting other parents and sharing experiences was mentioned by six participants, e.g., *“Knowing I’m not alone, sharing coping strategies”*. In relation to the facilitators, they were described as experienced, knowledgeable, professional, good listeners, non-judgemental and friendly (*N* = 4). With regard to those absent from the workshops, two participants spoke of the potential value of including other family members and friends of young voice hearers, e.g., *“The workshop made me reflect on how much we have needed to educate and dispel fear in friends, families and parents around our son.”*

### What Should the Workshop Space Look Like?

The second category related to how participants experienced workshop content and delivery and recommendations for how they would improve the experience. Participants stated workshop content was informative (*N* = 8) and helpful (*N* = 4) and gave them a better understating on the types of treatment their child could be offered for distressing voices (*N* = 4). Participants suggested improvements relating to in-person workshops (*N* = 5), individual sessions (*N* = 2), less formality (*N* = 2), more sessions to cover content (*N* = 2), no group work (*N* = 2), shorter sessions (*N* = 2), a slower pace (*N* = 1), increasing accessibility to people with differing technological abilities (*N* = 1), and delivery within school environments (*N* = 1).

### Outcomes

A descriptive summary, standardised effect size estimates (i.e., Cohen’s *d*_*av*_), and 95% confidence intervals of unstandardised effect estimates for outcomes at T0, T1 and T2 are displayed in Tables 3 and 4. Comparisons between sociodemographic characteristics and baseline outcomes between completers and non-completers indicated a significant difference between the age of completers (M_AGE_ = 49.60) and non-completers (M_AGE_ = 43.44), (*t* (17) = 2.95, *p* = .009) however, there were no other significant differences between groups. As such, missing data was assumed to be missing at random (MAR; Seaman et al., 2013).

**Table 3.** Descriptive Statistics and Standardised Effect Size Estimates for Self-Efficacy and ABVH Outcomes.

Outcome Variable	T0		T1		T2		T0-T1	T1-T2	T0-T2
	M (min-max)	SD	M (min-max)	SD	M (min- max)	SD	*Cohen’s d*_*av*_	*Cohen’s d*_*av*_	*Cohen’s d*_*av*_
Self-efficacy to ask a young person if they hear voices	76.90 (5–100)	29.18	75.21 (16–100)	23.34	73.78 (25, 100)	29.34	0.10	−0.19	−0.24
Self-efficacy to discuss voice-hearing with a young person	68.80 (3–100)	27.28	78.64 (25 – 100)	21.82	75.56 (35, 100)	25.90	0.38	−0.28	0.21
Self-efficacy to provide useful information about voice-hearing to a young person	30.90 (1–91)	27.00	75.99 (40 – 96)	17.93	65.44 (45, 100)	22.16	2.16	−0.73	1.67
ABVH	4.43 (2.80, 6.20)	0.74	5.37 (3.80, 6.90)	0.82	5.60 (5.00, 6.40)	0.47	1.18	0.30	1.82

**Table 4.** Unstandardised Effect Sizes and Associated 95% Confidence Intervals for T0-T1, T1-T2 and T0-T2.

Outcome Variable	T0-T1		T1-T2		T0-T2	
	Unstandardised Effect	95% CI	Unstandardised Effect	95% CI	Unstandardised Effect	95% CI
Self-efficacy to ask a young person if they hear voices	2.71	[-18.96, 24.39]	−4.02	[-25.14, 17.38]	1.28	[-32.79, 20.35]
Self-efficacy to discuss voice-hearing with a young person	9.71	[-9.05, 28.48]	−5.54	[-25.21, 15.88]	11.86	[-25.30, 35.52]
Self-efficacy to provide useful information about voice-hearing to a young person	44.93	[24.06, 59.80]	−12.76	[-20.72, −0.84]	38.24	[17.67, 54.33]
ABVH	0.96	[0.40, 1.53]	0.14	[-0.61, 0.88]	1.08	[0.63, 1.52]

#### Self-Efficacy

There was little change in participants’ self-efficacy to ask a young person if they hear voices between T0-T1 (*d*_*av*_ = 0.10) and a small decrease was found between T0-T2 (*d*_*av*_ = −0.24). Small increases in self-efficacy to discuss voice hearing with a young person were noted between T0-T1 (*d*_*av*_ = 0.38) and T0-T2 (*d*_*av*_ = 0.21). The largest change was found in relation to increased self-efficacy to provide useful information about voice-hearing to a young person between T0-T1 (*d*_*av*_ = 2.16) and between T0-T2 (*d*_*av*_ = 1.67).

#### ABVH

There were improvements in attitudes and beliefs following the workshop; participants had more positive attitudes and beliefs towards voice hearing between T0-T1 (*d*_av_ = 1.18) and these improvements were maintained at follow-up (T0-T2; *d*_av_ = 1.82), both with large effect sizes.

## Discussion

This study assessed the acceptability of, and outcomes associated with, a psychoeducational workshop for parents of young people experiencing hearing voices. Twenty-one participants were recruited to the study, 15 attended the workshops and 10 offered full datasets. These high levels of attrition may reflect the demands upon the time of parents and other common barriers noted in previous research such as accommodating work schedules and childcare (Finigan-Carr et al., 2014).

The workshop was experienced as acceptable and satisfactory by the majority of parents. The qualitative data offered insights into the people that parents felt should be included within the workshop space, and what the workshop space should look like. The benefits of group participation with peers, such as feeling less alone, and hearing and sharing experiences with others, was recognised in this study, similar to findings documented in the literature by both voice hearers and the parents of people who hear voices (Goodliffe et al., 2010; Petrakis et al., 2014). Parents also reflected on who was missing from the workshop and suggested including other family members and friends. This implies that a more inclusive approach may be needed when involving members of a young person’s support network.

Suggestions for changes to the workshops included: in-person workshops, individual sessions, less formality, more sessions, no group work, shorter sessions, a slower pace of sessions, increasing accessibility to people with differing technological abilities, and delivery within school environments. Conflicting experiences between parents highlighted how a ‘one size fits all’ approach did not appear to be possible. Whilst service providers typically recognise the importance of person-centred approaches within healthcare, service-level constraints of shortage of resources have previously been identified as a barrier to this (Gondek et al., 2017). Collaboration with people with lived experience on adaptations to the workshop should be considered to tailor the offering to a wider audience of parents and individuals within the young person’s support network.

Outcomes suggested benefits in relation to improving parents’ self-efficacy to provide useful information about voice hearing to a young person and of having more positive attitudes and beliefs towards voice hearing. Baseline ratings indicated that parents already had confidence in asking about and discussing voice hearing with a young person. This finding replicated that found by Rammou (2021) amongst CAMHS clinicians working with young voice hearers. When considering the ongoing focus of interventions for parents, they seem to require information to take into conversations with young people, as opposed to needing support to begin a conversation in the first place. Indeed, it is important to be mindful that this may not be true for all parents, such as those who are not already engaged with CAMHS services. Further research is needed to explore the needs of parents at different stages in their journey of supporting a young person.

This study responded to calls from parents for interventions which can help them to support young voice hearers, and the study was conducted within the routine clinical practice environment of the NHS, therefore the sample were likely representative of those who would normally access NHS clinical services. It’s important to acknowledge that organisations outside of the NHS can offer support to the parents of young people who are distressed by hearing voices (e.g., Voice Collective), and these parents may differ from the participants within this study in terms of demographics and needs.

There are a number of limitations that warrant consideration. First, the outcome measures lacked psychometric validation and reliability. Although the self-efficacy and ABVH measures were found to be valid when used with clinicians, this was the first time they had been used with parents. The internal reliability statistics for the ABVH outcome (0.66) was below a minimum acceptable threshold of 0.70 (Tavakol & Dennick, 2011). Given such measurement issues, the findings from the study need to be interpreted with caution and further research is needed to establish the internal and external validity and improve the reliability of these outcome measures. Second, the small study sample size was compounded by high attrition rates. Findings from small sample sizes can lead to overinflations of the size of the effect of an intervention and research suggests that attrition rates above 20% can introduce bias and be a threat to validity (Grimes & Schulz, 2002). Third, receipt of and engagement with the ‘Overcoming Distressing Voices’ book may have influenced the T2 outcomes. Within a future study, the book could be sent to parents after the completion of data collection. Finally, participants in the sample were predominantly White British (89.5%), with English as their first language (94.7%), consistent with the demographics of the Southeast of England (Office for National Statistics, 2020). The lack of cultural diversity of the sample limits the generalisability of findings to those from culturally diverse backgrounds, where interpretation and explanations for hearing voices may differ (Luhrmann et al., 2015). Future studies should aim to be inclusive of participants from a range of cultural backgrounds. Attention should also be paid to inclusion in relation to digital access and literacy.

The study highlights several directions for future research. Future development of the workshop would benefit from being guided by collaborative conversations with young people and key stakeholders such as family members, clinicians, and wider networks (i.e., schools). Consideration could also be given to the inclusion of friends within the workshop, as support from friends can play an important role in adolescence (Friedlmeier and Granqvist, 2006). Young people who hear voices report limited social connections with others, and report lower social support from friends (Rammou, 2021), possibly due to lack of disclosure resulting from a fear of perceived stigma and/or lack of interpersonal trust (Birchwood, 2003; Mortenson, 2009). Whilst disclosing voices to friends might be difficult for young voice hearers, providing opportunities for young people to involve friends within interventions may normalise hearing voices and add to the emerging literature on peer interventions in early psychosis (Harrop et al., 2015; White et al., 2017; Nguyen et al., 2022). Future research might also usefully explore the promotion of voice hearing information in school environments to increase the reach of support offered to young people and be inclusive of those who do not access mental health services (Kapur et al., 2014; Parry & Varese, 2021a), perhaps through Mental Health Support Team (MHST) practitioners. Finally, consideration should be given to the more extensive involvement of people with lived experence (including family and friends) in the design and delivery of future studies.

In conclusion, a psychoeducational workshop for parents of young voice hearers was seen as acceptable to many parents who attended and has the potential to increase parental confidence to provide useful information to young voice hearers, whilst providing a space for parents to feel less alone in their family’s experience. Whilst this workshop responds to a gap in the literature for increased involvement of parents in young people’s mental health care, other social supports, and delivery settings, such as friends and schools, should be considered within future developments to maximise benefits to young voice hearers and support them to cope with their voice hearing experiences.
